# Evaluation of the Visual Analog Score (VAS) to Assess Acute Mountain Sickness (AMS) in a Hypobaric Chamber

**DOI:** 10.1371/journal.pone.0113376

**Published:** 2014-11-18

**Authors:** Jialin Wu, Yu Chen, Yongjun Luo

**Affiliations:** 1 Battalion 8 of Cadet Brigade; Third Military Medical University, Chongqing, PR China; 2 Department of Military Medical Geography; College of High Altitude Military Medicine, Third Military Medical University, Chongqing, PR China; 3 Key Laboratory of High Altitude Medicine (Ministry of Education), Third Military Medical University, Chongqing, PR China; Texas Tech University Health Science Centers, United States of America

## Abstract

**Objective:**

The visual analog score (VAS) is widely used in clinical medicine to evaluate the severity of subjective symptoms. There is substantial literature on the application of the VAS in medicine, especially in measuring pain, nausea, fatigue, and sleep quality. Hypobaric chambers are utilized to test and exercise the anaerobic endurance of athletes. To this end, we evaluated the degree of AMS using the visual analog scale (VAS) in a hypobaric chamber in which the equivalent altitude was increased from 300 to 3500 m.

**Methods:**

We observed 32 healthy young men in the hypobaric chamber (Guizhou, China) and increased the altitude from 300 to 3500 m. During the five hours of testing, we measured the resting blood oxygen saturation (SaO_2_) and heart rate (HR). Using the VAS, we recorded the subjects' ratings of their AMS symptom intensity that occurred throughout the phase of increasing altitude at 300 m, 1500 m, 2000 m, 2500 m, 3000 m, and 3500 m.

**Results:**

During the phase of increasing altitude in the hypobaric chamber, the patients' SaO_2_ was 96.8±0.8% at 300 m and 87.5±4.1% at 3500 m (P<0.05) and their HR was 79.0±8.0 beats/minute at 300 m and 79.3±11.3 beats/minute at 3500 m. The incidence of symptoms significantly increased from 21.9% at an altitude of 1000 m to 65.6% at an altitude of 3500 m (P<0.05). The composite VAS score, which rated the occurrence of four symptoms (headache, dizziness, fatigue, and gastrointestinal discomfort), was significantly correlated with elevation (P<0.01).

**Conclusion:**

Based on the experimental data, the VAS can be used as an auxiliary diagnostic method of Lake Louise score to evaluate AMS and can show the changing severity of symptoms during the process of increased elevation in a hypobaric chamber; it also reflects a significant correlation with altitude.

## Introduction

Acute mountain sickness (AMS) is a series of acute hypoxic events that occur with the transition from the plains to a plateau or from a plateau to a higher altitude plateau over a short period of time [1]. The common symptoms are nausea, headache, anorexia, insomnia, fatigue/lassitude, vomiting and dizziness [2]–[4]. The rate and height of the ascent and the type of transportation used to ascend are determinants of the incidence of AMS [5]. Due to the limitations of the physical conditions, little research can be completed at the plateau. Therefore, hypobaric chambers are used to simulate the low-oxygen environment of the plateau for scientific research [6]–[8]. A hypobaric chamber is a small room that is not completely closed and relies on suction to create the hypobaric hypoxic state within the cabin. The equivalent altitude can be adjusted as needed to several kilometers or tens of thousands of meters above sea level to simulate a hypoxic environment [6]–[8]. Hypobaric chambers are utilized to test and exercise the anaerobic endurance of athletes, and many researchers have used them to explore AMS [9].

Many studies have used the Lake Louise score (LLS) and the Environmental Symptoms Questionnaire (ESQ-III) to assess AMS in the clinic and in research. The LLS consists of a self-evaluation questionnaire and a normative clinical assessment [10], [11], which evaluates the 5 symptoms of headache, dizziness, fatigue, gastrointestinal discomfort, and sleep. It is widely used by expedition leaders to assess AMS as they must often determine whether a climber can continue hiking [12]. The ESQ-III was developed by the United States military in the late 1970s and early 1980s [13], [14], and it includes 11 items to assess AMS [15]. The visual analog score (VAS) is another scoring system that has been suggested for assessing AMS based on its usefulness in performing other clinical evaluations [16]. Many studies have shown various applications of the VAS in medicine, and the VAS is commonly used to measure fatigue, hunger, satiety [17], headache [18], [19], nausea, pain, and sleep quality [20], [21]. It has also been used to measure objective and subjective cognition [22]–[24]. The VAS reduces the reliance on written language [20], [25]. Subjects only need to make a mark on a 10-cm horizontal line; the left end of the line is 0 mm, which indicates a lack of symptoms, and the right end is 100 mm, which indicates that the symptoms are serious. Intermediate points along the line represent varying degrees of symptoms. Subjects make a mark on this line to indicate the degree of symptoms they are experiencing. A symptom intensity from 0 to 4 mm represents almost no symptoms; an intensity from 5 to 44 mm represents a mild level of symptoms; an intensity from 45 to 74 mm represents a moderate level of symptoms; and an intensity from 75 to 100 mm represents a severe level of symptoms [26].

Currently, there are some studies on the use of the VAS to assess AMS symptoms. Additionally, the VAS is used as an auxiliary diagnostic method for the LLS or Environmental Symptoms Questionnaire (ESQ-III). In previous studies, some researchers have required that both the LLS and the ESQ-III be used to define AMS. However, as the qualifications of AMS have different definitions, Roach et al. stated that a headache with an LLS≥3 as one of the symptoms could indicate AMS [27], and Sampson et al. stated that an ESQ-III≥0.7 could indicate AMS [11]. Furthermore, some researchers consider an LLS≥5 to be necessary for the diagnosis of AMS [28], [29]. The VAS score corresponding to AMS as defined by LLS and ESQ-III also has different definitions. Wagner et al. determined that a VAS score of 16 mm indicates AMS, which corresponds to LLS = 5 and ESQ-III = 0.7 [30]. Hext et al. reported cut-off points of 22 mm, representing an LLS≥3, and 33 mm, representing an ESQ-III = 0.7 [31]. There is currently no agreement over the specific VAS score that should be used to evaluate AMS. Therefore, the purpose of this study was to use the VAS to assess the incidence of AMS in 32 subjects who remained in a hypobaric chamber from 300 m to 3500 m above sea level.

## Materials and Methods

### Study setting

We observed 32 healthy young men in a hypobaric chamber during a 5-hour period over which the altitude increased from 300 to 3500 m and then decreased from 3500 to 300 m. The ascending stage lasted for three hours, and the hypobaric chamber altitude was increased by 500 m every 20 minutes each time, except for the first adjustment, which went directly from 300 to 1000 m. After each adjustment, there was a 10 minute period for pressure equilibration. The descending stage lasted two hours, and the altitude was reduced by 500 m every 15 minutes with a 10 minute period for pressure equilibration; the final pressure adjustment went directly from 1500 to 300 m.

### Data collection

Prior to the test, we measured the participants' resting blood oxygen saturation (SaO_2_) and heart rate (HR) using a Tuffsat pulse oximeter, and we determined the subjects' basic information using a questionnaire. In the ascent and descent phases, we measured the subjects' SaO_2_ and HR at 1500 m, 2000 m, 2500 m, 3000 m, and 3500 m using the Tuffsat pulse oximeter. The intensity of the subjects' AMS symptoms was measured using the VAS in the ascent phase at the altitudes of 300 m, 1000 m, 1500 m, 2000 m, 2500 m, 3000 m, and 3500 m. The symptoms included headache, dizziness, fatigue, and gastrointestinal discomfort. For every elevation gradient, we gave each participant a VAS questionnaire, which had four 10-cm horizontal lines that corresponded to headache, dizziness, fatigue, and gastrointestinal discomfort. They marked a single slash in the corresponding position of the questionnaire that best represents their worst feeling for the four symptoms. Study materials were available in Chinese, and volunteers completed the questionnaire according to the grade of their more severe symptom. The left end of the line is 0 mm with the word “none”, and the right end is 100 mm with the word “severe”. Intermediate points along the line represent varying degrees of symptoms. Then, we transformed the corresponding tag into a score. Because the test time was short and the condition of the hypobaric chamber was inappropriate for sleep, we did not assess the symptoms of sleep disorder. Throughout the experimental process, the subjects did not perform any strenuous exercise, and all measurements and survey questions were carried out while the subject was sitting. This study was approved by the ethical committee of the Third Military Medical University in China.

### Statistical analyses

Two investigators (Jialin Wu and Yu Chen) measured the distance in millimeters from the left end of the line to the slash mark. Utilizing SPSS version 19.0, we performed repeated measure ANOVA between the VAS score and altitude. We defined AMS as headache and LLS≥3, and this definition has been shown to determine the climbers' degree of AMS [32]. Additionally, on the basis of the study by Hext et al., we used cut-off points of the VAS ≥22 mm, representing an LLS ≥3. So, we classified participants as AMS present (VAS ≥22 mm) or AMS not present (VAS <2 mm), which is shown in the scatter diagram. The homogeneity test of variance (F test) was used for the data analysis of SaO_2_ and HR. The correlation between the incidence of the four symptoms, the total number of symptoms and elevation was analyzed using the Pearson correlation coefficient analysis. All tests of significance were two-tailed, P<0.05 was considered significant and P<0.01 was considered very significant.

## Results

### Volunteer characteristics

Thirty-two volunteers consented to the study, and all of them returned questionnaires with the outcome data. Their ages ranged from 19 to 25 years with an average age of 21.6±1.85 years. A total of 15 lived in southwest China (46.9%), and 17 were from the non-southwest region (53.1%). Thirty (93.8%) were of Han ethnicity, one was Manchu (3.1%), and another was of Hui ethnicity (3.1%; Table 1).

**Table 1:** Baseline characteristics of the volunteers.

Variable	Mean or proportion
Age (yr)	21.6±1.85
Gender (% male)	100%
Language (%)	
Mandarin	100%
Ethnicity (%)	
Han	93.80%
Manchu	3.10%
Hui	3.10%
Smoking (%)	
Yes	15.60%
No	84.40%
Wohnort (%)	
Southwest China	46.90%
Non-southwest China	53.10%

### Changes in the SaO_2_ during the ascent and descent phases

**Figure 1:**
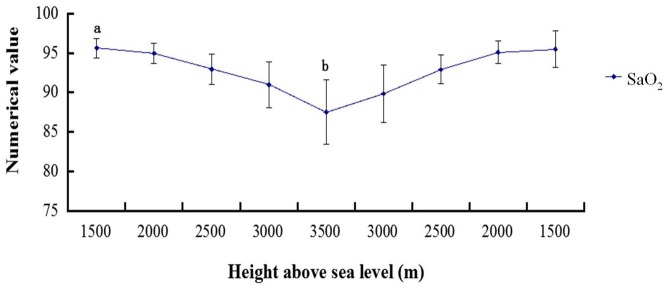
Graph showing the SaO_2_ during the ascent and descent phases. a P<0.01: compared with the altitudes of 2500 m, 3000 m, and 3500 m; b P<0.01: compared with the altitudes of 3000 m, 2500 m, 2000 m, and 1500 m.

The highest SaO_2_ was 96.8±0.79%, which occurred at 300 m. With increasing altitude, the SaO_2_ gradually decreased. The lowest SaO_2_ was 87.5±4.1%, which occurred at an altitude of 3500 m. We compared SaO_2_ levels at 1500 m with those at 2500 m, 3000 m, and 3500 m (P<0.01), and there was a significant correlation. During the descent phase, the SaO_2_ rose with decreasing altitude to 95.5±2.3% at 1500 m. Comparing SaO_2_ level at 3500 m with those at 3000 m, 2500 m, 2000 m, and 1500 m (P<0.01), there was a significant correlation (Fig. 1).

### Changes in the HR during the ascent and descent phases

**Figure 2:**
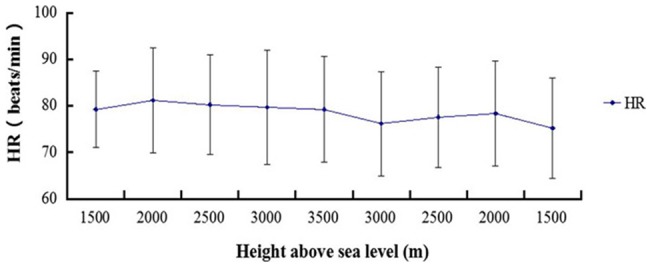
Graph showing the HR during the ascent and descent phases. The change in HR during the ascent and descent phases had no significant relationship with altitude changes (P>0.05).

Over the course of the altitude increase, the participants' heart rate gradually increased. The maximum heart rate was 81.2±11.3 beats/minute at 2000 m, which decreased to 79.3±11.3 beats/minute when the altitude was 3500 m. During the descent phase, the heart rate decreased, falling to 75.2±10.8 beats/minute at an altitude of 1500 m (Fig. 2). The F test of HR during the ascent and descent phases did not demonstrate significant differences (P>0.05).

### Changes in the incidence of symptoms associated with increasing altitude

Throughout the experiment, some people experienced only one symptom and some experienced a few symptoms; others experienced no symptoms. At an altitude of 1000 m, a total of 7 subjects experienced symptoms of AMS, including dizziness (35.7%), fatigue (28.6%), gastrointestinal discomfort (21.4%), and headache (14.3%). At an altitude of 1500 m, 6 subjects experienced acute symptoms of AMS, and dizziness was still the most common symptom, occurring in 30.8% of the subjects. At an altitude of 2000 m, 11 subjects experienced symptoms of acute altitude sickness, including primarily headaches and dizziness, both of which were observed in 27.8% of the subjects. With an increase in altitude to 2500 m above sea level, the number of subjects experiencing symptoms of acute mountain sickness rose to a total of 15; fewer experienced headaches, but the proportion of subjects experiencing dizziness rose to 37.5%. At 3000 m above sea level, 18 subjects had symptoms of acute mountain sickness, including dizziness (40%) and fatigue (33.3%). Finally, at an altitude of 3500 m, a total of 21 subjects had symptoms of acute mountain sickness, including headache (41.0%), fatigue (30.8%), gastrointestinal discomfort (15.4%) and dizziness (12.8%; Table 2).

**Table 2:** The incidence of symptoms associated with increasing altitude (n = 32).

Symptoms	300 m	1000 m	1500 m	2000 m	2500 m	3000 m	3500 m
Total number of subjects	0	7(21.9%)	6(18.8%)	11(34.4%)	15(46.9%)	18(56.3%)	21(65.6%)
Headache	0	2(14.3%)	3(23.1%)	5(27.8%)	3(12.5%)	3(10.0%)	5(12.8%)
Dizziness	0	5(35.7%)	4(30.8%)	5(27.8%)	9(37.5%)	12(40.0%)	16(41.0%)
Fatigue	0	4(28.6%)	3(23.1%)	4(22.2%)	7(29.2%)	10(33.3%)	12(30.8%)
Gastrointestinal discomfort	0	3(21.4%)	3(23.1%)	4(22.2%)	5(20.8%)	5(16.7%)	6(15.4%)

We evaluated the incidence rate of the four symptoms in different altitudes and the occurrence rate of total number of symptoms. The Pearson correlation coefficient analysis revealed the incidence of four symptoms and the total number had a varying degree of correlation with elevation. Headache was significantly correlated with altitude (P<0.05). Dizziness, Fatigue, Gastrointestinal discomfort and total incidence had a tremendous significant correlation with altitude (P<0.01) (Table 3).

**Table 3:** The incidence rate of different symptom at different altitudes.

Elevation (m)	Symptoms and incidence rate (%)				
	Headache	Dizziness	Fatigue	Gastrointestinal discomfort	Total incidence
300	0	0	0	0	0
1000	6.25% (2/32)	15.6% (5/32)	12.5% (4/32)	9.38% (3/32)	21.94% (7/32)
1500	9.38% (3/32)	12.5% (4/32)	9.38% (3/32)	9.38% (3/32)	18.8% (6/32)
2000	15.6% (5/32)	15.6% (5/32)	12.5% (4/32)	12.5% (4/32)	34.4% (11/32)
2500	9.38% (3/32)	28.1% (9/32)	21.94% (7/32)	15.6% (5/32)	46.9% (15/32)
3000	9.38% (3/32)	37.5% (12/32)	32.0% (10/32)	15.6% (5/32)	56.3% (18/32)
3500	15.6% (5/32)^a^	50.0% (16/32)^b^	37.5% (12/32)^c^	18.8% (6/32)^d^	65.6% (21/32)^e^

a p<0.05: compared with altitudes of 300 m, 1000 m, 1500 m, 2000 m, 2500 m, and 3000 m.

b p<0.01: compared with altitudes of 300 m, 1000 m, 1500 m, 2000 m, 2500 m, and 3000 m.

c p<0.01: compared with altitudes of 300 m, 1000 m, 1500 m, 2000 m, 2500 m, and 3000 m.

d p<0.01: compared with altitudes of 300 m, 1000 m, 1500 m, 2000 m, 2500 m, and 3000 m.

e p<0.01: compared with altitudes of 300 m, 1000 m, 1500 m, 2000 m, 2500 m, and 3000 m.

### The subjective score of symptoms in the ascent phase as assessed by the VAS

During the ascent phase, the number of subjects experiencing the symptoms increased, but the fractional value of the VAS for each subjective severity of the symptoms was always 20 or less (0 to 20). At an altitude of 1000 m, 2 people had a headache with an intensity of 10 mm. At 3500 m above sea level, a total of 5 subjects suffered headache symptoms, ranging in VAS score from 5 to 10 mm. A total of 5 subjects suffered dizziness at an altitude of 1000 m; 4 of them rated the dizziness at 10 mm and another at 20 mm. When the altitude increased to 3500 m, a total of 15 subjects had symptoms of dizziness; 4 rated the dizziness at 20 mm, and the rest rated it at 10 mm. Four subjects experienced fatigue symptoms at an altitude of 1000 m; 3 rated the fatigue at 10 mm, and another rated it at 20 mm. At an altitude of 3500 m, the number of subjects experiencing fatigue increased to 10; most rated the fatigue at 10 mm, and 2 rated it at 20 mm. Gastrointestinal discomfort at an altitude of 1000 m was experienced by only 3 people, who rated it at 10 mm; at 3500 m, the number of subjects with gastrointestinal discomfort increased to 6, but the rating remained at 10 mm (Table 4). These results suggest that the change in the score of the symptoms was not very obvious in the hypobaric chamber; the symptoms were all under the mild level.

**Table 4:** The VAS score of symptoms (n = 32).

Symptoms	Scores	300 m	1000 m	1500 m	2000 m	2500 m	3000 m	3500 m
Headache	5	0	0	0	0	0	0	1 (3.1%)
	10	0	2 (6.3%)	3 (9.4%)	5 (15.6%)	3 (9.4%)	3 (9.4%)	4 (12.5%)
	20	0	0	0	0	0	0	0
	30–100	0	0	0	0	0	0	0
Dizziness	5	0	0	0	0	0	0	1 (3.1%)
	10	0	4 (12.5%)	3 (9.4%)	5 (15.6%)	9 (28.1%)	11 (34.4%)	11 (34.4%)
	20	0	1 (3.1%)	1 (3.1%)	0	0	1 (3.1%)	4 (12.5%)
	30–100	0	0	0	0	0	0	0
Fatigue	5	0	0	0	1 (3.1%)	1 (3.1%)	0	0
	10	0	3 (9.4%)	3 (9.4%)	3 (9.4%)	5 (15.6%)	7 (21.9%)	10 (31.2%)
	20	0	1 (3.1%)	0	0	1 (3.1%)	2 (6.3%)	2 (6.3%)
	30–100	0	0	0	0	0	0	0
Gastrointestinal discomfort	5	0	0	0	0	0	0	0
	10	0	3 (9.4%)	3 (9.4%)	4 (12.5%)	5 (15.6%)	5 (15.6%)	6 (18.8%)
	20	0	0	0	0	0	0	0
	30–100	0	0	0	0	0	0	0

### Repeated measure ANOVA between the altitude and VAS score

**Figure 3:**
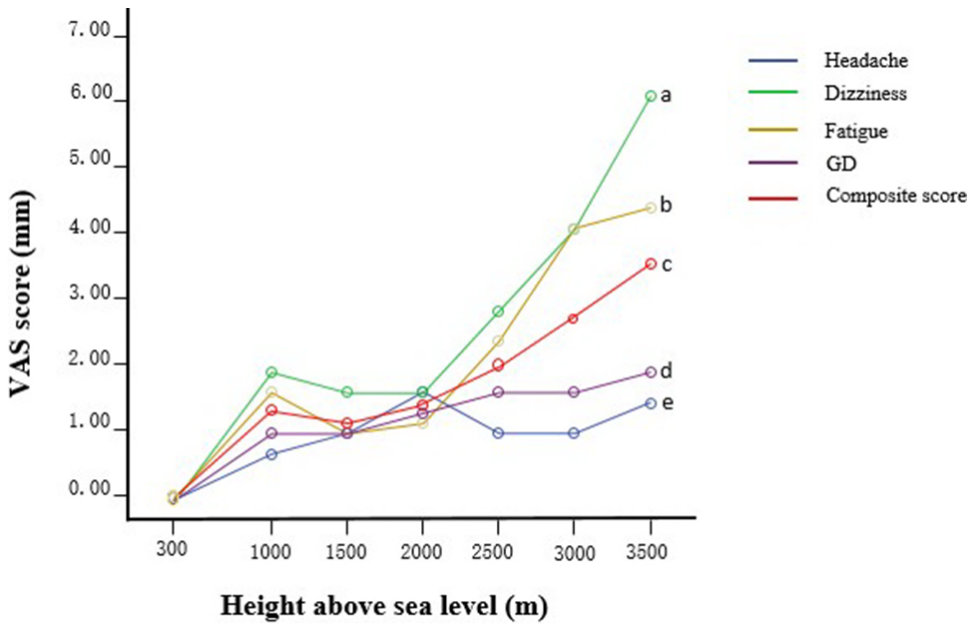
Repeated measure ANOVA between the altitude and VAS score. GD: Gastrointestinal discomfort. There was no correlation between headache and gastrointestinal discomfort with altitude (^e^ P>0.05, ^d^ P>0.05). Fatigue had a significant interaction with altitude (^b^ P<0.05). The correlation between dizziness and the composite score with altitude was very significant (^a^ P<0.01, ^c^ P<0.01).

The composite score is the overall score of the four symptoms (headache, dizziness, fatigue and gastrointestinal discomfort). Repeated measure ANOVA displays the correlation between the four symptoms and composite score, which was assessed by the VAS with increasing elevation. There was no correlation between headache and gastrointestinal discomfort with altitude (^e^ P>0.05, ^d^ P>0.05). Fatigue showed a significant difference with altitude (^b^ P<0.05). Comparing the dizziness and composite score at 3500 m with those at 300 m, 1000 m, 1500 m, 2000 m, 2500 m and 3000 m (^a^ P<0.01, ^c^ P<0.01), revealed a significant correlation with altitude (Fig. 3).

### Scatter diagram of the VAS at different elevations

**Figure 4:**
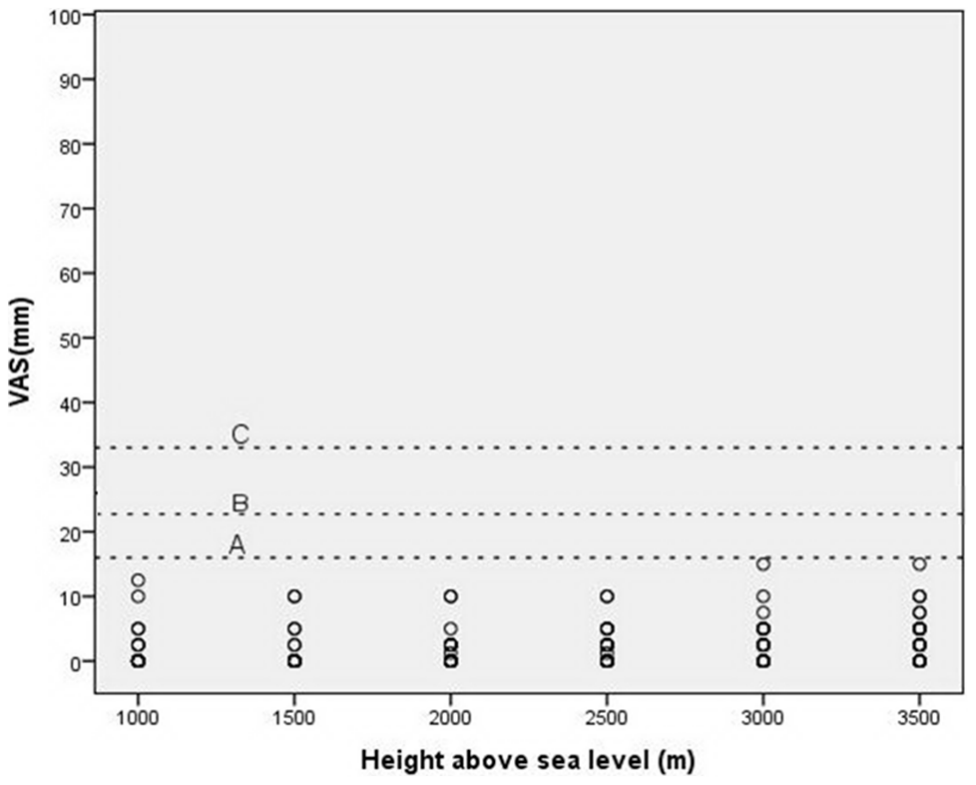
Scatter diagram of the visual analog scale (VAS). Relationship between altitude and average VAS score. Dotted line A represents a score of 16 mm for the VAS; dotted line B = 22 mm and dotted line C = 33 mm.

The scatter diagram of the VAS score for all subjects contains the composite of the four symptoms. Dotted line A represents the VAS criterion scores of 16 mm; dotted lines B and C represent 22 mm and 33 mm, respectively (Fig. 4). Most of the scores were approximately 0, and the highest score was 15 mm. Dotted line B represents the VAS score of 22 mm, which was determined by Hext et al. to identify AMS and corresponds to LLS≥3. Therefore, none of the subjects suffered from AMS, and only one subject obtained a score of 15 mm at 3500 m. Of course, 15 mm is also below the other standards, which are 16 mm and 33 mm. Therefore, it is clear that, in this study, assessing AMS in the hypobaric chamber using the VAS score did not achieve the expected results.

## Discussion

In this study, the SaO_2_ of the subjects decreased with an increase in elevation in the hypobaric chamber. At an altitude of 300 m, SaO_2_ was 96.8±0.8%, and at 3500 m, the SaO_2_ was only 87.5±4.1% (Fig. 1). In a hypobaric chamber, as the altitude increases, the air pressure in the cabin and the oxygen partial pressure decrease. Atmospheric pressure is inversely related to altitude because the level of oxygen at high altitude is decreased [33]. The decreased partial pressure of oxygen reduces the ability of hemoglobin to carry oxygen in the blood and for oxygen to dissociate from HbO_2_. Therefore, there is a lack of oxygen in the body, and hypoxia occurs. As a result, the partial pressure of oxygen, the product of the fractional concentration of oxygen and the overall pressure, is the key factor in AMS [33]. Additionally, peripheral chemoreceptors (located in the carotid and aortic bodies) can sense the decreased PO_2_ or increased PCO_2_ in the arterial blood. These impulses will be transmitted to the medulla oblongata by the sinus and vagus nerves. This stimulation causes the heart rate to increase, but this mechanism of adjustment only occurs in severe hypoxia and does not under normal circumstances [34], [35]. Similarly, the data analysis showed that HR was not significantly different (P>0.05, Fig. 2) during the ascent and descent phases. After all, the SaO_2_ decline was not very intense in the entire experiment and did not reach the degree of hypoxia or asphyxia.

In general, during the experiment, the incidence of subjects who developed the symptoms of AMS increased along with the increasing altitude, from 21.9% at an altitude of 1000 m to 65.6% at an altitude of 3500 m (The incidence rate of different altitudes is show in Table 3, P<0.01). Therefore, with increasing altitude in the hypobaric chamber, both the air pressure and the oxygen content decreased, and the incidence of AMS gradually increased. During the experiment, all subjects were instructed to not use any medicine or supplemental oxygen to prevent the occurrence of AMS.

By using the VAS to quantify the severity of subjective symptoms of AMS in this experiment, we were able to show that the intensity of the symptoms was weak at low altitude (1000 m); all of the symptoms were rated as 10 mm or less at that altitude (Table 4). The intensity of the symptoms increased with increasing altitude to scores of predominantly 10 or 20 mm (3500 m, the symptoms of dizziness and fatigue were particularly prevalent). The scores for all of these symptoms fall under the range of the mild level (5–44 mm) on the VAS. Based on repeated measure ANOVA, the composite VAS score was significantly correlated with altitude (P<0.01; Fig. 3). These showed that the change in the intensity of the symptoms was not very obvious in the hypobaric chamber; all symptoms were mild or lower. Such a low score differs from those of previous studies [36]–[38]. Our study was performed in a hypobaric chamber, and the previous research was conducted in the real plateau environment.

The VAS score in the scatter diagram is the average of four symptoms because sleep was not included (Fig. 4). Dotted line B represents the VAS score of 22 mm, which was the minimum score defining the occurrence of AMS. However, the data indicated that none of the participants reached that score and suffered AMS. In other studies, the incidence of AMS was obviously different [39]. Vardy J et al. reported that the incidence of AMS was 10% from 3000 to 4000 m [38], and Honigman et al. reported 25% at 6300 to 9700 feet [36]. Hence, assessing AMS in the hypobaric chamber with the VAS score in this study did not achieve the expected results. Of course, the other groups' research was conducted in a real plateau environment, and we evaluated subjects in a hypobaric chamber. Nevertheless, the participants experienced symptoms in the hypobaric chamber. Even so, as a tool for the continuous measurement of symptoms, the VAS is highly suitable for the detection of symptom changes over time [22], [37]. The advantage of the VAS is the visual nature of the data; furthermore, trends and changes can easily be discovered and interpreted [31].

There were some limitations to this experiment. The entire experiment was limited to five hours in duration, the elevation increase was not extreme (3500 m was the maximum elevation), and the subjects may not have fully developed all the symptoms of AMS. As a result, the symptoms of AMS were not very apparent. Additionally, the sample size was small; larger samples from other populations would be preferable for further evaluating AMS with the VAS in the future.

## Conclusions

This study evaluated the incidence of AMS in a hypobaric chamber using the VAS. The experimental data explicitly informed us about the changes in the incidence rate of symptoms in the entire group. Based on the experimental data, the VAS can be used as an auxiliary diagnostic method for the LLS and can show the changing severity of symptoms in the process of increased elevation in a hypobaric chamber, and the VAS scores show a significant correlation to altitude.

## Supporting Information

Table S1 **Volunteer characteristics.**
(DOCX)

Table S2 **The ascent and descent phases.**
(DOCX)

Table S3 **VAS score at different altitude.**
(DOCX)
