# Supplementary material for: Evaluation of the Visual Analog Score (VAS) to Assess Acute Mountain Sickness (AMS) in a Hypobaric Chamber
Source: PLoS One. 2014 Nov 18;9(11):e113376. doi: 10.1371/journal.pone.0113376 (PMC4236192; doi:10.1371/journal.pone.0113376)
Supplement: Table S1 — Volunteer characteristics. (DOCX) [file pone.0113376.s001.docx]

**Table S1. Volunteer characteristics**

| Number | Language | Gender | Ethnicity | Age(yr) | Wohnort | Smoking |
| --- | --- | --- | --- | --- | --- | --- |
| 1 | Mandarin | male | Hui | 24 | Henan | No |
| 2 | Mandarin | male | Han | 21 | Sichuan | Yes |
| 3 | Mandarin | male | Han | 21 | Guizhou | No |
| 4 | Mandarin | male | Han | 23 | Shandong | Yes |
| 5 | Mandarin | male | Han | 21 | Xinjiang | No |
| 6 | Mandarin | male | Han | 26 | Anhui | No |
| 7 | Mandarin | male | Han | 20 | Chongqing | No |
| 8 | Mandarin | male | Han | 19 | Sichuan | No |
| 9 | Mandarin | male | Han | 23 | Chongqing | No |
| 10 | Mandarin | male | Han | 23 | Jiangsu | No |
| 11 | Mandarin | male | Han | 23 | Chongqing | Yes |
| 12 | Mandarin | male | Han | 22 | Sichuan | No |
| 13 | Mandarin | male | Han | 20 | Gansu | No |
| 14 | Mandarin | male | Han | 20 | Sichuan | No |
| 15 | Mandarin | male | Han | 24 | Shaanxi | Yes |
| 16 | Mandarin | male | Han | 21 | Xinjiang | No |
| 17 | Mandarin | male | Han | 21 | Zhejiang | No |
| 18 | Mandarin | male | Han | 21 | Shaanxi | No |
| 19 | Mandarin | male | Han | 19 | Guizhou | No |
| 20 | Mandarin | male | Han | 20 | Sichuan | No |
| 21 | Mandarin | male | Han | 23 | Henan | No |
| 22 | Mandarin | male | Han | 20 | Guizhou | No |
| 23 | Mandarin | male | Han | 20 | Yunnan | No |
| 24 | Mandarin | male | Han | 19 | Sichuan | No |
| 25 | Mandarin | male | Han | 21 | Sichuan | No |
| 26 | Mandarin | male | Han | 22 | Hubei | No |
| 27 | Mandarin | male | Han | 22 | Shandong | No |
| 28 | Mandarin | male | Han | 21 | Jiangsu | No |
| 29 | Mandarin | male | Han | 20 | Guizhou | No |
| 30 | Mandarin | male | Han | 25 | Henan | Yes |
| 31 | Mandarin | male | Han | 21 | Shandong | No |
| 32 | Mandarin | male | Manchu | 25 | Liaoning | No |
