# Supplementary material for: Evaluation of the Visual Analog Score (VAS) to Assess Acute Mountain Sickness (AMS) in a Hypobaric Chamber
Source: PLoS One. 2014 Nov 18;9(11):e113376. doi: 10.1371/journal.pone.0113376 (PMC4236192; doi:10.1371/journal.pone.0113376)
Supplement: Table S2 — The ascent and descent phases. (DOCX) [file pone.0113376.s002.docx]

**Table S2. the ascent and descent phases**

| **Elevation** | **300 m** | | **1500 m** | | **2000 m** | | **2500 m** | | **3000 m** | | **3500 m** | | **3000 m** | | **2500 m** | | **2000 m** | | **1500 m** | |
| --- | --- | --- | --- | --- | --- | --- | --- | --- | --- | --- | --- | --- | --- | --- | --- | --- | --- | --- | --- | --- |
|  | **SaO2(%)** | **HR (beats/minute)** | **SaO2(%)** | **HR (beats/minute)** | **SaO2(%)** | **HR (beats/minute)** | **SaO2(%)** | **HR (beats/minute)** | **SaO2(%)** | **HR (beats/minute)** | **SaO2(%)** | **HR (beats/minute)** | **SaO2(%)** | **HR (beats/minute)** | **SaO2(%)** | **HR (beats/minute)** | **SaO2(%)** | **HR (beats/minute)** | **SaO2(%)** | **HR (beats/minute)** |
| 1 | 97 | 75 | 96 | 76 | 96 | 93 | 95 | 70 | 95 | 71 | 87 | 77 | 92 | 78 | 94 | 69 | 95 | 77 | 96 | 77 |
| 2 | 98 | 82 | 96 | 79 | 98 | 75 | 95 | 80 | 96 | 75 | 95 | 84 | 98 | 77 | 95 | 67 | 99 | 76 | 98 | 71 |
| 3 | 96 | 83 | 96 | 77 | 96 | 77 | 95 | 84 | 94 | 83 | 89 | 91 | 93 | 83 | 89 | 81 | 95 | 86 | 96 | 90 |
| 4 | 97 | 74 | 96 | 81 | 96 | 72 | 91 | 78 | 93 | 76 | 93 | 72 | 93 | 62 | 95 | 64 | 96 | 64 | 97 | 61 |
| 5 | 96 | 88 | 92 | 84 | 95 | 84 | 95 | 95 | 91 | 81 | 91 | 86 | 95 | 76 | 92 | 82 | 91 | 73 | 96 | 65 |
| 6 | 96 | 103 | 96 | 82 | 96 | 85 | 93 | 88 |  |  | 91 | 80 |  |  | 94 | 69 | 96 | 76 | 98 | 77 |
| 7 | 96 | 79 | 96 | 88 | 94 | 87 | 93 | 93 | 90 | 87 | 86 | 93 | 88 | 88 | 93 | 89 | 95 | 77 | 95 | 85 |
| 8 | 97 | 78 | 96 | 81 | 94 | 88 | 91 | 78 | 88 | 83 | 88 | 91 | 88 | 89 | 93 | 90 | 95 | 84 | 97 | 78 |
| 9 | 96 | 87 | 96 | 89 | 92 | 94 | 92 | 86 | 90 | 96 | 80 | 86 | 84 | 91 | 91 | 87 | 94 | 88 | 96 | 90 |
| 10 | 97 | 62 | 96 | 70 | 95 | 74 | 91 | 80 | 92 | 72 | 83 | 74 | 90 | 65 | 92 | 65 | 96 | 75 | 95 | 70 |
| 11 | 96 | 72 | 95 | 66 | 95 | 77 | 94 | 73 | 93 | 65 | 86 | 66 | 93 | 63 | 94 | 66 | 95 | 62 | 96 | 60 |
| 12 | 95 | 81 | 94 | 74 | 94 | 76 | 92 | 86 | 88 | 84 | 81 | 89 | 88 | 81 | 91 | 99 | 95 | 85 | 95 | 93 |
| 13 | 98 | 71 | 95 | 66 | 94 | 77 | 92 | 63 | 90 | 59 | 79 | 64 | 85 | 61 | 94 | 61 | 96 | 61 | 96 | 62 |
| 14 | 96 | 64 | 96 | 72 | 94 | 83 | 92 | 69 | 87 | 68 | 85 | 61 | 90 | 76 |  |  | 96 | 79 |  |  |
| 15 | 98 | 82 | 96 | 84 | 94 | 88 | 92 | 88 | 91 | 88 | 90 | 89 | 93 | 80 | 94 | 90 | 96 | 82 | 96 | 86 |
| 16 | 98 | 80 | 96 | 91 | 97 | 107 | 95 | 95 | 96 | 99 | 92 | 100 | 93 | 96 | 96 | 90 | 96 | 90 | 96 | 91 |
| 17 | 97 | 76 | 96 | 87 | 95 | 93 | 90 | 93 | 93 | 91 | 81 | 90 | 83 | 87 | 89 | 93 |  |  |  |  |
| 18 | 97 | 96 | 95 | 100 | 94 | 98 | 88 | 93 | 83 | 92 | 85 | 90 | 90 | 93 | 94 | 87 | 94 | 94 | 95 | 80 |
| 19 | 97 | 80 | 97 | 87 | 97 | 66 | 95 | 65 | 92 | 63 | 88 | 71 | 90 | 70 | 93 | 81 | 95 | 86 | 96 | 75 |
| 20 | 97 | 73 | 96 | 77 | 96 | 90 | 96 | 81 | 89 | 97 | 89 | 78 | 92 | 71 | 93 | 74 | 96 | 73 | 95 | 72 |
| 21 | 96 | 72 | 95 | 73 | 94 | 79 | 91 | 72 | 86 | 75 | 86 | 75 | 90 | 77 | 96 | 69 | 93 | 65 | 96 | 64 |
| 22 | 97 | 74 | 96 | 76 | 95 | 72 | 94 | 82 | 92 | 87 | 85 | 77 | 88 | 72 | 93 | 88 | 95 | 84 | 96 | 78 |
| 23 | 96 | 88 | 97 | 83 | 96 | 85 | 93 | 81 | 93 | 86 | 88 | 90 | 85 | 95 | 91 | 87 | 93 | 85 | 95 | 82 |
| 24 | 96 | 87 | 95 | 82 | 95 | 90 | 95 | 94 | 94 | 102 | 91 | 97 | 92 | 83 | 94 | 84 | 95 | 94 | 95 | 84 |
| 25 | 98 | 64 | 95 | 67 | 96 | 64 | 92 | 85 | 92 | 56 | 88 | 64 | 92 | 58 | 94 | 61 | 96 | 66 | 85 | 56 |
| 26 | 98 | 74 | 98 | 74 | 96 | 64 | 96 | 74 | 91 | 72 | 92 | 69 | 88 | 71 | 91 | 74 | 97 | 68 | 97 | 63 |
| 27 | 97 | 79 | 95 | 74 | 95 | 70 | 92 | 59 | 91 | 70 | 91 | 63 | 90 | 60 | 95 | 62 | 95 | 64 | 96 | 68 |
| 28 | 97 | 72 | 97 | 73 | 95 | 64 | 95 | 67 | 91 | 60 | 88 | 60 | 93 | 60 | 93 | 67 | 95 | 61 | 97 | 61 |
| 29 | 96 | 88 | 95 | 82 | 94 | 89 | 94 | 75 | 87 | 82 | 93 | 70 | 83 | 75 | 92 | 77 | 95 | 85 | 96 | 87 |
| 30 | 97 | 73 | 97 | 67 | 95 | 60 | 93 | 60 | 92 | 73 | 88 | 74 | 92 | 62 | 90 | 77 |  |  | 92 | 72 |
| 31 | 97 | 88 | 96 | 87 | 94 | 93 | 92 | 89 | 89 | 93 | 90 | 77 | 90 | 78 | 92 | 80 | 94 | 106 |  |  |
| 32 | 97 | 82 | 92 | 88 | 92 | 84 | 91 | 92 | 92 | 84 | 82 | 89 | 84 | 84 | 93 | 76 | 94 | 86 | 95 | 84 |
